# A Novel Dual-Energy CT Method for Detection and Differentiation of Intracerebral Hemorrhage From Contrast Extravasation in Stroke Patients After Endovascular Thrombectomy: Feasibility and First Results

**DOI:** 10.1007/s00062-022-01198-3

**Published:** 2022-08-12

**Authors:** Risto Grkovski, Leyla Acu, Uzeyir Ahmadli, Robert Terziev, Tilman Schubert, Susanne Wegener, Zsolt Kulcsar, Shakir Husain, Hatem Alkadhi, Sebastian Winklhofer

**Affiliations:** 1 Department of Neuroradiology, Clinical Neuroscience Cente, University Hospital Zurich, University of Zurich, Zurich, Switzerland; 2 Faculty of Medicine, University Of Ljubljana, Ljubljana, Slovenia; 3 Department of Radiology, University Medical Centre Maribor, Maribor, Slovenia; 4 Department of Neurology, Clinical Neuroscience Cente, University Hospital Zurich, University of Zurich, Zurich, Switzerland; 5 Institute of Diagnostic and Interventional Radiology, University Hospital Zurich, University of Zurich, Zurich, Switzerland

**Keywords:** Spectral computed tomography, Cerebrovascular accident, Endovascular treatment, Brain hemorrhage, Contrast extravasation

## Abstract

**Purpose:**

Dual-energy computed tomography (DECT) has been shown to be able to differentiate between intracranial hemorrhage (ICH) and extravasation of iodinated contrast media (contrast staining [CS]). TwinSpiral DECT is a recently introduced technique, which allows image acquisition at two different energy levels in two consecutive spiral scans. The aim of this study was to evaluate the feasibility and accuracy of TwinSpiral DECT to distinguish between ICH and CS after endovascular thrombectomy (EVT) in patients with acute ischemic stroke.

**Methods:**

This retrospective single-center study conducted between November 2019 and July 2020 included non-contrast TwinSpiral DECT scans (tube voltages 80 and 150Sn kVp) of 39 ischemic stroke patients (18 females, 21 males, mean age 69 ± 11 years) within 48–72 h after endovascular thrombectomy. Parenchymal hyperdensity was assessed for the presence of ICH or/and CS by two board certified and fellowship-trained, blinded and independent neuroradiologists using standard mixed images and virtual non-contrast (VNC) images with corresponding iodine maps from TwinSpiral DECT. Follow-up examinations (FU; CT or MRI) were used as a standard of reference. Sensitivity, specificity, and accuracy for the detection of ICH as well as the inter-reader agreement were calculated.

**Results:**

Parenchymal hyperdensities were detected in 17/39 (44%) patients. Using DECT, they were classified by both readers as ICH in 9 (53%), CS in 8 (47%), and mixture of both in 6 (35%) cases with excellent agreement (κ = 0.81, *P* < 0.0001). The sensitivity, specificity, and accuracy for the detection of ICH in DECT was 90% (95% confidence interval [CI]: 84–96%), 100% (95% CI 94–100%) and 95% (95% CI 89–100%), and in mixed images 90% (95% CI 84–96%), 86% (95% CI 80–92%) and 88% (95% CI 82–94%), respectively. Inter-reader agreement for detecting ICH on DECT compared to the mixed images was κ = 1.00 (*P* < 0.0001) vs. κ = 0.51 (*P* = 0.034).

**Conclusion:**

TwinSpiral DECT demonstrates high accuracy and excellent specificity for differentiating ICH from CS in patients after mechanical thrombectomy due to acute ischemic stroke, and improves inter-reader agreement for detecting ICH compared to the standard mixed images.

## Introduction

Endovascular thrombectomy (EVT) has become an indispensable treatment in selected patients with an acute ischemic stroke due to a large vessel occlusion.

Standard of care follow-up imaging is, among others, performed to rule out intracranial hemorrhage (ICH) and is recommended within 24 h after EVT, before administering anticoagulant or antiplatelet agents. Contrast staining (CS) is a frequently found phenomena after EVT in ischemic brain tissue due to a leakage of the blood brain barrier (contrast extravasation during the EVT). Unfortunately, CS might look very similar to the ICH in a conventional single-energy computed tomography (SECT) and might result in diagnostic uncertainty, misdiagnosis, and possibly the wrong treatment for the patient. Recent clinical studies have shown an importance of an early diagnosis of post-EVT hemorrhage, intraparenchymal or subarachnoid, and its impact on patient’s management and outcome [1, 2], thus more affordable and accessible techniques for improved hemorrhage detection are needed.

Dual-energy CT (DECT) is an advanced CT technique, which adds value based on the different x‑ray attenuation properties of tissue at high and low energy x‑rays. The two acquired datasets (low and high kV) from DECT enable material decomposition due to the unique high and low energy linear attenuation coefficients for a given material. Based on these characteristics, a three-material decomposition algorithm enables the differentiation between various materials with high atomic numbers, e.g. iodine, blood, or calcium [3–6].

Several clinical applications of DECT have been introduced in the field of neuroradiology [7], among them the generation of virtual non-contrast images and corresponding color encoded iodine maps. This approach allows removal of all iodine-containing structures from prior intravenous or intra-arterial iodine applications of an image (virtual non-contrast [VNC]) and to emphasize all iodine-containing structures (iodine maps). By using this method, previous studies have shown the ability of DECT to differentiate between ICH and CS after EVT [8, 9], as well as demonstrating the clinical value of this technique in imaging of patients with an acute ischemic stroke regarding decision making, outcome, prognosis and risk of ICH [1, 10].

TwinSpiral DECT, a version of the dual spiral technology, is a recently introduced DECT technique for acquiring datasets at two different energy levels for spectral separation. First, a low kV scan is acquired, immediately followed by a high kV tin filtered second scan, resembling a single scan by minimizing the delay between the two scans. To the best of our knowledge, no previous study investigated this technique for neuroradiological applications.

The aim of this study was to evaluate the feasibility and accuracy of TwinSpiral DECT to distinguish between hemorrhage and iodine extravasation after mechanical thrombectomy in patients with an acute ischemic stroke.

## Material and Methods

### Patient Selection

All procedures were performed in accordance with local and federal regulations and the Declaration of Helsinki. The retrospective study was approved by the local ethics committee (BASEC-Nr. 2018-01212).

In this retrospective cohort study, 39 patients (21 males, 18 females, mean age 69 ± 11 years, range 45–90 years) with a TwinSpiral DECT scan as follow-up after EVT in patients with acute ischemic stroke were included. Scans were performed between November 2019 and July 2020. Patients were identified through the institutional radiology information system with the search terms “dual-energy” and “mechanical thrombectomy” in the radiology reports. Inclusion criteria were the availability of TwinSpiral DECT images within 24 h after EVT and at least one additional follow-up examination within 48–72 h after the TwinSpiral CT scan, either done by a CT with a non-contrast phase, or a magnetic resonance imaging (MRI) with a susceptibility weighted imaging (SWI).

### CT Data Acquisition

**Fig. 1:**
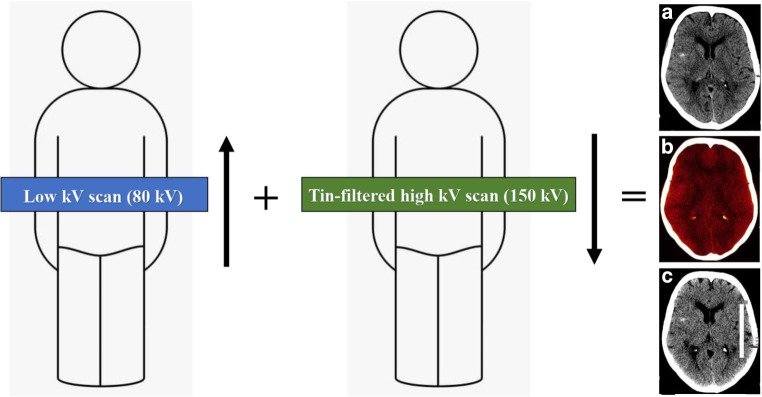
Schematic mode of operation of a single-source TwinSpiral dual-energy CT: Two consecutive scans at a different energies (low and a high kV scan) are performed directly one after the other. From these two datasets, conventional mixed CT images (**a**), as well as dedicated reconstructions, such as color-coded iodine overlay images (**b**) and virtual non-contrast (VNC) images (**c**) can be postprocessed

A head CT imaging was performed using a single-source CT scanner (Somatom X.cite, Siemens Healthcare, Erlangen, Germany) in TwinSpiral dual-energy mode. Tube voltages were set to 80 and 150 kVp, the latter operated with tin (Sn) filtration, and with corresponding quality reference tube current-time products of 220 and 179 mAs, respectively, using automated tube current modulation (CAREDose4D) (Fig. 1). Further scanning parameters were as follows: slice acquisition, 2 × 0.6 × 64 mm by means of a z-flying focal spot; rotation time, 1.0 s; and pitch, 0.55. The mean volume CT dose index (CTDI_vol_) of the protocol was 43.7 ± 3.4 mGy.

### Postprocessing

All steps are part of the routine clinical protocol in DECT after mechanical thrombectomy at our department. Postprocessing and reconstruction was performed on a dedicated workstation (syngo MultiModality Workplace, CT Dual-Energy, Brain Hemorrhage, syngo.via, version VB.40 client 4.0, Siemens). The following postprocessing steps were semi-automatically performed by the scanner integrated software. First, conventional mixed CT images were reconstructed with a weighting factor of 0.5, representing a mix of images from the low-energy and high-energy scan (medium-smooth kernel, Hr40s) resembling single-energy CT [11, 12]. Second, color-coded iodine overlay images and VNC images were calculated from DECT TwinSpiral data by using the brain hemorrhage pre-set [4]. Image reconstruction of mixed, iodine maps, and VNC images was performed identically in an axial orientation with a slice thickness of 4 mm and increment of 4 mm. The underlying technical background for the differentiation of ICH and CS by DECT was described in detail elsewhere [13]. Briefly, an advanced three material decomposition algorithm was applied, which allows the differentiation of iodine, hemorrhage, and brain parenchyma.

After postprocessing all image data was transferred to our hospital’s picture archiving and communication system (Agfa, Version 6.6.1, Mortsel, Belgium).

### Image Analysis

Two experienced and fellowship trained neuroradiologists performed the image analysis. The readout was carried out on high-resolution monitors (Flexscan MX 210, Eizo, Ishikawa, Japan) using the picture archiving and communication system (IMPAX EE R20, XVIII SU1, Agfa Healthcare, Bonn, Germany) of the hospital.

First, the image quality of standard mixed images and VNC and iodine maps reconstructions from TwinSpiral (VNC and iodine maps) was qualitatively rated as diagnostic or non-diagnostic regarding the evaluation of the brain parenchyma. Second, standard mixed and DECT images were evaluated regarding the presence of suspicious hyperdensities (yes/no, represents either CS or ICH) and further assessed based on the presence on iodine images (indicating CS) or on VNC images (representing ICH). In case of both hyperdensities in iodine maps and VNC images, a combination of CS and ICH was postulated. Third, with a time interval of 2 weeks, follow-up examinations (either non-contrast CT or MRI with SWI sequences were evaluated) as proof of evidence, as shown in previous studies [14]. CS in non-contrast follow-up CT was defined as a full or near complete washout of the hyperdensities detected in the initial CT scan after mechanical thrombectomy. ICH in a non-contrast follow-up CT was defined as a hyperdensity, which remained stable or near stable compared to the initial CT and if a typical ICH related perifocal edema was visible. ICH in MRI was defined as a susceptibility artifacts in SWI images [6, 15, 16]. Consensus reading was performed in case of discrepancies between readers. Both readers were blinded to the images or image reports of each of the previous or next steps of the image analysis and to the results of each other. After a period of more than 8 weeks, a re-readout of the scans by reader 1 for the purpose of intra-reader agreement was performed.

### Statistical Analysis

Continuous variables were defined as mean ± standard deviation (SD) or median and range. Categorical variables were defined as frequencies and percentages.

Cohen κ coefficients were calculated to evaluate the inter-rater agreement regarding the classification of cases as ICH, CS or mixture of both using DECT images, as well as correctly detected ICH on DECT compared to the standard mixed images. κ values between 0.41 and 0.75 were interpreted as fair to good, and values between 0.75 and 1 were interpreted as excellent, according to criteria originally proposed by Landis and Koch [17].

Sensitivity, specificity, and accuracy for the correct assessment of the presence or absence of ICH were calculated. In cases of a combination of ICH and CS, sensitivity, specificity, and accuracy statistical calculations included these patients in the ICH group. Confidence intervals (CI) were computed at a level of 95%. Due to the small number of evaluated cases, exact confidence intervals based on binomial probabilities were calculated [18]. Follow-up images were used as a reference standard.

## Results

**Fig. 2:**
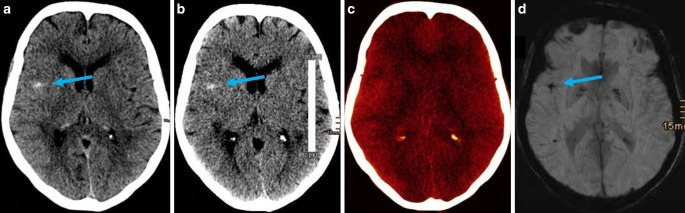
A standard native non-contrast computed tomography (CT) mixed image from TwinSpiral dual-energy CT shows a hyperdensity in the Sylvian cortex on the right side (*blue arrow*) in an acute ischemic stroke patient after endovascular thrombectomy (**a**). The hyperdensity is also seen in the same location on the virtual non-contrast (VNC) image (**b**). No hyperdensity is seen on the iodine map (**c**). Follow-up magnetic resonance image (MRI) (**d**) showing a susceptibility artifact (*blue arrow*) on a susceptibility-weighted imaging (SWI) corresponding to the hyperdense area seen on the standard mixed and VNC images, but not on the iodine map, thus proving an intracerebral hemorrhage

**Fig. 3:**
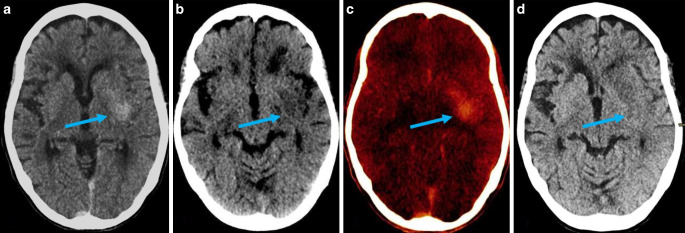
A hyperdensity is seen in the left basal ganglia (*blue arrow*) on a standard non-contrast mixed image from TwinSpiral dual-energy CT image in an acute ischemic stroke patient after endovascular thrombectomy (**a**). No hyperdensity is seen in the same location on the VNC image (**b**). Hyperdensity is seen on the iodine map (**c**). Follow-up non-contrast CT (**d**) showing no hyperdensity at the corresponding location (*blue arrow*), thus indicating the contrast extravasation resorbed in follow-up imaging

A diagnostic image quality was found in all 39 (100%) patients for both mixed images and iodine and VNC reconstructions from TwinSpiral DECT in both readers (Figs. 2 and 3).

Hyperdensities were detected in 17 of the 39 cases (44%) in mixed and VNC images after EVT (κ = 1.00, *P* < 0.001). Using DECT (VNC and iodine maps), they were classified as ICH in 9 (53%), CS in 8 (47%), and a mixture of both in 6 (35%) cases with excellent agreement between readers (κ = 0.81, *P* < 0.0001).

**Fig. 4:**
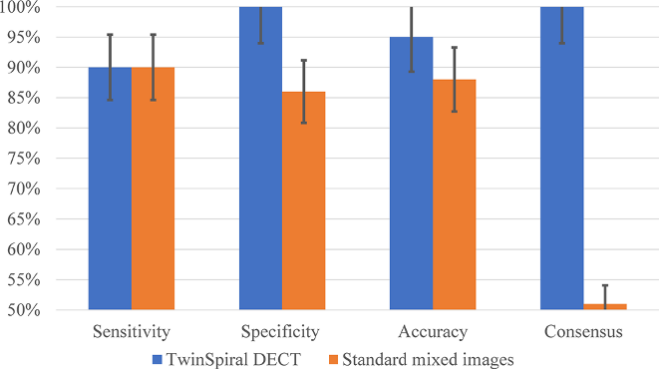
Performance of the TwinSpiral DECT compared to the standard mixed images for detecting an ICH

For the TwinSpiral DECT the sensitivity, specificity, and accuracy for the correct detection of ICH using VNC and iodine maps were 90% (95% CI 84–96%), 100% (95% CI 94%–100%) and 95% (95% CI 89–100%), respectively. On the mixed images these same parameters resulted in respective values of 90% (95% CI 84–96%), 86% (95% CI 80–92%) and 88% (95% CI 82–94%) for both readers. Inter-reader agreement between the two readers for detecting ICH on DECT compared to the mixed images was κ = 1.00 (*P* < 0.0001) vs. κ = 0.51 (*P* = 0.034) (Fig. 4). Intra-reader agreement of reader 1 for detecting ICH on DECT and mixed images was κ = 1.00 and κ = 0.86 (*P* < 0.0001), respectively.

## Discussion

This study introduces a novel dual-energy CT technique for the differentiation of CS from ICH after EVT in patients with acute stroke due to a large vessel occlusion. It indicates that the TwinSpiral DECT is feasible to separate these two entities and allows to clarify this clinically relevant question after mechanical thrombectomy. A high sensitivity, specificity, and accuracy for the presence of ICH in a non-contrast DECT suggests that this technique may be used in a clinical routine. The results of the study further indicate that the TwinSpiral DECT technique can be used as an alternative to the previously available DETC techniques, such as dual-source, rapid kilovoltage-switching, or dual-layer DECT.

To achieve different energies in a single acquisition, various DECT techniques have been introduced into a clinical imaging in recent years: a dual-source DECT with two x‑ray sources rotating simultaneously around the patient; a twin beam DECT with a filter-based splitting of the x‑ray beam, a rapid kilovoltage-switching DECT with a gemstone scintillator detector (GSI), a dual-layer detector-based (sandwich detector) spectral DECT technique and a photon-counting CT [19]. This requires dedicated CT scanners with either single or dual source, dual layer, or gemstone ability. The TwinSpiral technique, however, seems to be technically simpler. A tin filter provides a powerful spectral separation, while a stellar detector enables high-quality imaging at a temporal resolution in low-dose or low-signal imaging, thus providing low-dose DECT scans. Along with utilization of Recon&GO, this enables advanced comprehensive and automated routine-ready solution, well suited for departments with single-energetic scanners for less costs than dual-energetic scans, thus making it more accessible for a broader range of hospitals and providing potential cost-benefit.

A number of previous studies have shown benefits in utilization of DECT in stroke imaging protocols. This includes various aspects, such as differentiation of ICH and CS by using a dual-source DECT approach [9, 20], improved diagnosis and classification of ICH after EVT with a fast kVp-switching CT technique [1], hemorrhagic transformation after endovascular thrombectomy with potential to affect future antithrombotic strategy [21], quantitative assessment of the maximum iodine concentration within the suspicious hyperdensities [22], intracranial hemorrhage prediction [23], improved histological composition of a thrombus in an in vitro study [24] and a better visualization of early ischemic changes of brain tissue after mechanical thrombectomy [25], in addition to further applications in the field of neuroradiology, which include the differentiation between tumor bleeding and pure hemorrhage, or the automated bone removal in DECT angiography [8, 26–28]. At our department, the DECT is fully integrated in our routine clinical workflow after mechanical thrombectomy. The decision whether follow-up examinations require CT or MRI depends on the individual situation of each patient and on the scanner availability.

One of the drawbacks of the TwinSpiral technique could be its susceptibility regarding the patient motion. In case the patient moves between the low kV and the high kV scan, this potentially would result in a complete loss of the DECT information; however, the morphological information could still be retrieved e.g. from the low KV scan, even though a minor loss of quality can be expected here. In our series of TwinSpiral scans in acute stroke patients after mechanical thrombectomy, all scans demonstrated a diagnostic image quality. None of the patients demonstrated relevant motion between the low and the high kV scans.

We found two cases in where the parenchymal hyperdensities were classified as CS according to the DECT, but which demonstrated ICH in the follow-up MRI. In both patients, it was also retrospectively not possible to define with certainty whether bleeding was already present during the first follow-up examination, or whether it was a secondary hemorrhagic transformation or reperfusion hemorrhage, which occurred between the DECT and the follow-up imaging. Interestingly, in both cases the follow-up examination was made by MRI using SWI sequences, where tiny susceptibility artifacts were visible and therefore were interpreted as an ICH in our study. In such cases, it is difficult to accurately assess the diagnostic value of DECT due to the time difference in regard to the control imaging, in which additional changes may have occurred; however, it would be interesting to see how DECT can improve risk assessment of hemorrhagic transformation of ischemic territories, where CS is present.

We acknowledge the following study limitations. First, the number of patients included in this work was relatively low and additional studies with a higher number of cases are required to confirm our results. Second, the readers could not have been completely blinded, as the VNC and iodine map images could have been recognized due to their distinctive characteristics when evaluated by the DECT experienced readers. Third, we were not able to compare the TwinSpiral DECT to previous techniques, such as dual-source or rapid kilovoltage-switching. Finally, follow-up imaging was not performed at predefined time intervals and using the same imaging technique.

## Conclusion

The VNC and iodine images derived from the TwinSpiral DECT enable an accurate detection of ICH after mechanical thrombectomy in patients with acute ischemic stroke. CS can be reliably differentiated from ICH which might have an impact on the prognosis, outcome and further therapeutic management of the patient.
